# Marburg Virus Re-Emerged in 2022: Recently Detected in Ghana, another Zoonotic Pathogen Coming Up Amid Rising Cases of Monkeypox and Ongoing Covid-19 Pandemic- Global Health Concerns and Counteracting Measures

**DOI:** 10.1080/01652176.2022.2116501

**Published:** 2022-08-22

**Authors:** Ranjit Sah, Aroop Mohanty, Abdullah Reda, Abdelmonem Siddiq, Ranjan K. Mohapatra, Kuldeep Dhama

**Affiliations:** a Tribhuvan University Teaching Hospital, Institute of Medicine, Kathmandu, Nepal;;; b Harvard Medical School, Boston, MA, USA;;; c All India Institute of Medical Sciences, Gorakhpur, India;;; d Faculty of Medicine, Al-Azhar University, Cairo, Egypt;;; e Faculty of Pharmacy, Mansoura University, Egypt;;; f Department of Chemistry, Government College of Engineering, Keonjhar-758002, Odisha, India;;; g Division of Pathology, ICAR-Indian Veterinary Research Institute (IVRI), Izatnagar- 243122, India

Marburg virus (MARV), a highly pathogenic virus, the cause of a deadly disease Marburg virus disease (MVD), has recently been in news during mid of July 2022 owing to its outbreak in Ghana, an African country, wherein two infected persons died (Hussain, 2022; WHO, 2022a; Zhao et al., 2022). More recently, the third death due to MVD has been reported in Ghana (Reuters, 2022). MARV is an enveloped single-stranded RNA virus of the *Filovirus* genus belonging to the Filoviridae family that also contains the Ebola virus responsible for Ebola virus disease (EVD). EVD and MVD have both prompted health agencies to be on alert and implement national and regional emergency responses from time to time (Adepoju, 2021). Marburg Virus Disease (MVD), formerly known as Marburg hemorrhagic fever (MHF), is a very deadly and fatal illness in humans and non-human primates (NHPs). According to the Center for Disease Control and Prevention (CDC), Atlanta, GA, USA, the virus was first detected in the city Marburg (Germany) in August 1967 when 31 cases of MVD were reported and caused an outbreak (Feldmann et al. 1996; Bharat, et al. 2011). The name Marburg originated from this city and thus the virus was named as Marburg Virus (MARV). It was thought that the MARV infection might have been transmitted to Germany through Uganda by importation of infected African green monkey (*Cercopithecus aethiops*) tissue for poliomyelitis vaccine development. MARV is believed to be the first filovirus discovered (Siegert et al. 1968].

Over the past five decades, MVD outbreaks have occurred mainly in Africa, however its outbreaks have also been reported twice in Europe (Asad et al., 2020; Ristanović et al. 2020; Abir et al. 2022; WHO, 2022a; Zhao et al. 2022). Since its detection in 1967, the virus has mostly affected the African countries with first cases being seen in Zimbabwe in 1975 (3 cases) and Kenya in 1980 and 1987 (3 cases). A single isolated case was also seen in Russia due to a laboratory accident in 1994 (Nikiforov et al. 1994). The worst affected countries were the Democratic Republic of Congo (DPR) and Angola where 128 and 227 deaths were reported in 2000 and 2004, respectively (Colebunders et al. 2007; Towner et al. 2006). Few sporadic cases also appeared in between in other different countries with the first case in USA in 2008, who was identified as a USA citizen travelling from Uganda. A preliminary retrospective serological survey performed on 2,430 serum samples (obtained between 1997 and 2012) revealed a seroprevalence of 7.5 and 6.3% for MARV in Cameroon and Ghana, respectively, which suggested that filoviruses or related viruses were being circulated beyond the known endemic areas that might have remained undetected by existing surveillance efforts (Steffen et al., 2020). During August 2021, MVD outbreak has been reported from Republic of Guinea, West African country, amid the COVID-19 pandemic [Koundouno et al. 2022; Okonji et al., 2022].

**Figure 1:**
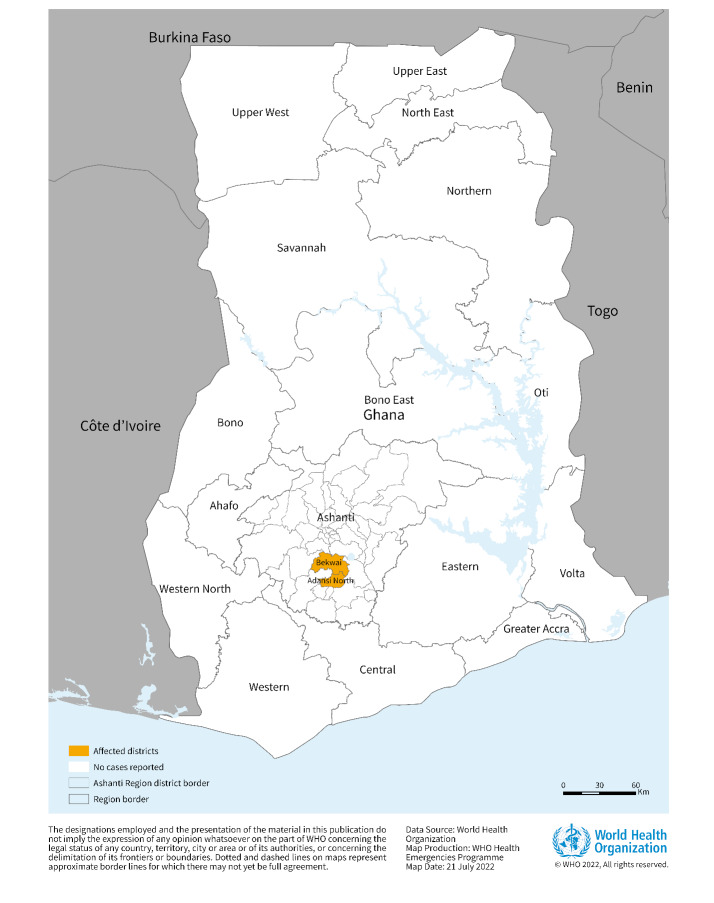
This Figure describes the geographical of the institutes that received the report, region of the two confirmed cases of Marburg virus disease reported in Ghana, as of 20 July 2022 (Adopted from WHO 2022b).

On July 17 2022, Ghana declared its first and West Africa’s second ever outbreak of MVD when it reported two cases to the health organization from the Southern Ashanti region as shown in Figure 1 (WHO 2022b). This region in Ghana is famous in the world for gold and cocoa production. Both cases were unrelated to each other. The first patient was a 26-year-old male who received care on 26 June and died the very next day whereas the second was a 51-year-old male who was admitted on 28 June and died on the same day itself. Both of them presented with similar symptoms including fever, diarrhea, nausea and vomiting. Immediately a team of World Health Organization (WHO) experts rushed to provide coordination, risk assessment, and infection control measures. As the outbreak was declared, swift action was taken, and more resources were employed to curtail the spread of the disease. Contact tracing was done and more than 90 contacts, including healthcare workers and community members were identified and were self-quarantined with continuous monitoring of symptoms. Of the 98 suspects, 39 have already completed their quarantine. As the cause and reason for the MARV infection in humans are unknown the frequent cross-border movements of people may lead further disease spread to the neighboring countries.

MVD is mainly thought to be an animal-borne zoonotic disease. The virus can be transmitted from bats to humans through prolonged exposure to mines and caves inhabited by bat colonies and via contact with bat saliva, faeces and contaminated fruits. In 2009, scientists successfully isolated MARV from healthy Egyptian fruit bats captured from a Uganda mine. *Rousettus aegyptiacus*, a Pteropodidae fruit bat, is the most common reservoir of MARV, and was detected in several patients in Kenya, Uganda, Gabon, South Africa, Sierra Leone and Zambia in between 2005-2018 (Abir et al. 2022). Kajihara et al. (2019) have detected MARV genome in Egyptian fruit bats captured in Zambia in September 2018 suggesting Zambia to be at risk for MVD. Phylogenetically, the virus is closely related to the viruses that caused MARV outbreaks previously in the DRC. It is therefore strongly suggested that fruit bats are the viral reservoir and natural host of MARV (Towner et al. 2009). Pawęska et al. (2020) have detected MARV RNA in the rectal swab samples of Egyptian rousette bats found in South Africa which suggests that the fecal contamination of such bat habitats will be a potential source of infection for humans and other animals. To determine the primary routes of virus shedding, Amman et al. (2015) have conducted an experiment on captive-bred *R. aegyptiacus* bats infected with MARV isolated from Egyptian fruit bat caught from Uganda where miners and tourists separately contracted MVD. The study showed routes of viral shedding capable of infecting humans and other animals. Amman et al. (2021) have also detected MARV RNA on the fruit in the food bowls of the experimentally infected bats. As most of the viral pathogens are of animal origins (particularly from various bat species) and infecting humans through cross-species transmission, the bat virus ecology and molecular biology has to be studied properly to understand the bat-borne viruses, and the current knowledge gaps to prepare for the next viral outbreak (Letko et al. 2020).

Human-to-human transmission can also occur through direct contact (broken skin or mucous membranes) with infected people's blood, secretions, or bodily fluids (Abir et al. 2022). MARV can also be transmitted through surfaces and materials (bed sheets, clothes) contaminated with these fluids and secretions. Viral transmission can happen during the preparation of an infected body for burial. Some assumptions state that it can also be transmitted sexually, as the virus has been detected in semen from a previously infected patient. **S**afer sex is suggested for MVD male survivors for ∼ 12 months, whereas semen must test negative. Along with *Hipposideros caffer* and some unclassified *Chiroptera,* they form the main reservoirs of infection. In addition, African green monkeys and pigs also play a role as potential amplifier hosts.

The disease can be divided into three distinct phases: the generalization phase, the early organ phase and the late organ/convalescence phase. The incubation period varies from 2-21 days, with the average duration being 5-9 days in humans (Kortepeter et al. 2020). It usually starts with unspecific symptoms including fever, headache, vomiting, diarrhea and severe myalgia. Muscle aches and pain are a common feature. These non-specific symptoms may hinder the diagnosis of the disease due to its similarity with other diseases such as malaria, typhoid, meningitis and other viral hemorrhagic fevers. Severe watery diarrhea, abdominal cramping and vomiting may be seen as early as the first week of illness. The look of patients at this phase has been described as showing “ghost-like” drawn features with deep-set eyes and expressionless faces. The second week is often characterized by severe hemorrhagic manifestations such as hematemesis, hematochezia, and spontaneous bleeding from venipuncture sites. During this phase there is development of a non-pruritic erythematous rash that starts to be focal initially but later on spread to different parts of the body and is considered common among MVD patients too (Martini 1971; Trappler et al. 1965). The severe phase of illness is marked by presence of high fever. It is often accompanied with confusion, irritability and aggression. The disease period involves certain abnormalities including disturbed temperature in between the elevation and demotion, leukopenia, lymphopenia, hypokalemia, thrombocytopenia and elevated liver enzymes. With the progression of the disease, the patient may experience kidney and multiorgan failure and shock with a case fatality rate (CFR) in between 23-90% (CDC 2020a; 2022; Duraffour et al. 2017; Bauer et al. 2019). The CFR might go as high as 100% in some cases.

As clinical diagnosis is difficult owing to the non-specificity of symptoms, laboratory confirmation is required in all cases. In case of a suspected patient, the first step is to isolate the patient and collect the samples maintaining utmost biosafety precautions, and wearing personal protective equipment (PPE) due to high biohazard risk. In case the sample needs to be transported, it should be packages using triple packaging system. RT-PCR, IgG-Capture ELISA, antigen capture detection tests and virus isolation by cell culture are conducted under maximum biological containment conditions (Biosafety Level – 4, BSL-4 laboratories) for the detection of the virus (CDC 2022b; CDC 2022c).

There is no specific vaccine or treatment available presently for Marburg virus. Treatment relies completely on supportive measures including rehydration, restoring lost blood and clotting factors, symptomatic treatment and checking complicating co-infections. However, there are a group of antivirals under development against the virus such as Galidesivir–BCX4430, Favipiravir–T-705, Remdesivir and Polyclonal concentrated IgG, which might protect against the disease (Kortepeter MG et al. 2020). Some of the promising vaccine platforms being utilized for developing MVD vaccines comprise of recombinant (r) vesicular stomatitis virus (VSV) vector MARV vaccine (rVSVΔG-MARV-GP), multi-epitope vaccine using vaccinomics and immunoinformatics approaches, proteome based designing of an epitope-based vaccine using computational approach, virus-like particles (VLP), replication-deficient simian adenoviral vector-based multi-filovirus vaccine, r-protein based filovirus multivalent vaccine, and rVSV-based vesiculovax vector based vaccine (rVSV-N4CT1-MARV-GP) (Hasan et al., 2019; Suschak et al., 2019; Dulin et al., 2021; Abir et al., 2022; Lehrer et al., 2021; Sami et al. 2021; Sebastian et al., 2020; Soltan et al., 2022; Woolsey et al. 2022; Zhao et al., 2022). In May 2020, the European Medicine agency (EMA) granted a marketing authorization to Zabdeno (Ad26.ZEBOV) and Mvabea (MVA-BN-Filo) against Ebola. The Mvabea contains a virus known as Vaccinia Ankara Bavarian Nordic (MVA) which has been modified to produce four proteins from Zaire ebolavirus and three other viruses of the same group. The vaccine might potentially protect against MVD, but its efficacy still has not been proven in clinical trials. More recently, a cloned rVSV-vectored Marburg vaccine candidate (PHV01) has been found to protect guinea pigs from lethal MVD, which is suggested to be suitable for making advances in developing vaccines to counteract MARV (Zhu et al. 2022). Treatment strategies being explored include antiviral drugs, small interfering RNA and small molecules, antibodies, monoclonal antibodies, cytokines, antisense, post-exposure vaccine, host-targeted and pan-filovirus therapeutics, and deep learning models-based identification of potent MARV inhibitors (Cross et al., 2018; Amatya et al., 2019; Abir et al., 2022; Bradfute 2022; Hickman et al., 2022; Kumari et al., 2022; Zhao et al., 2022).

Emerging infectious diseases markedly affect public health and the global economy. We still are not over with COVID-19 pandemic and now we have the episodes of rising cases of Monkeypox in several countries, spreading rapidly worldwide. The world must be on full alert regarding MARV as it is a highly contagious and deadly virus. If this outbreak goes out of hand, it will result in millions of deaths as it is a highly fatal disease with up to 90% mortality. There are a set of preventive measures recommended to prevent MVD. Although the exact preventive measures are not known there are certain measures that can be undertaken in order to interrupt the human-to-human transmission. Wearing of appropriate protective clothing and follow up of safety measures while visiting caves and mines inhabited by bats along with limiting the exposure to such animals is required. During such outbreaks, all animal products should be thoroughly cooked before consumption. Increasing awareness and educating the communities affected by MVD, both about the nature of disease and necessary containment measures will reduce the risk of transmission and spread. Any patient suspected or confirmed to have MVD must be isolated to undergo all relevant investigations. Also, all infection control measures should be strictly followed including mask-wearing, gowning by the healthcare professionals as they are more vulnerable to the disease and trying to provide all the available diagnostic tools to help the health authorities to confirm any suspected case (CDC 2022). As seen with Monkeypox, the disease travelled from Africa to the rest of the world; a similar feasible situation can happen with MVD. There is urgent need to screen all travellers from MARV endemic countries and also to develop rapid diagnostic tests to detect them before they carry the deadly virus to other countries.

MARV is a risk group 4 pathogen due to highly contagious nature and high CFR, therefore there is an urgent need to develop effective solutions against this deadly virus before it expands out widely from Africa to other countries (Soltan et al. 2022; Zhao et al., 2022; Zhao et al., 2022). MVD has been considered a neglected infectious disease, which has resulted into small outbreaks from time to time in past five decades, large outbreaks have been rare. However, being a highly contagious and fatal disease, it can possibly pose high global public health concern if it spreads in multiple countries owing to rapid international travel and globalization, climate changes and other risk factors. Immediate proactive control strategies and preparedness plans, enhancing surveillance, contact tracing, expanding of BSL-4 laboratories, adopting appropriate prevention and control measures including implementation of one health concept with multidisciplinary collaborative approaches would help in limiting the virus spread. There is need to in-depth understanding of MARV and disease (MVD) it causes, explore virus/host interactions, fill up the gaps in knowledge, and develop effective vaccines, antiviral drugs and therapies. Any feasible MARV spread to other countries amid the COVID-19 pandemic and rising cases of monkeypox, would create an immensely heavy burden on global health system and pose a high danger on the life of millions of people, hence we need to be fully ready and equipped in advance to counteract any feasible MVD outbreaks and/or spread of MARV in future.

**Acknowledgement**

All the authors acknowledge and thank their respective Institutes and Universities.

**Funding**

This is a compilation written by its authors and required no substantial funding to be stated.

**Disclosure statement**

All authors declare that there exist no commercial or financial relationships that could, in any way, lead to a potential conflict of interest.
